# Open Repair of Posterior Cruciate Ligament Tibial Bony Avulsion With Metal Anchor: A Case Report

**DOI:** 10.1155/2024/3137345

**Published:** 2024-07-09

**Authors:** Giovanni Bonaspetti, Stefano Tonolini, Giovanni Dib, Alessia Piovani

**Affiliations:** ^1^ Department of Orthopaedics and Trauma Surgery Clinical Institute S. Anna GSD-Istituto Clinico S. Anna GSD, Via del Franzone 31 25127, Brescia, Italy; ^2^ Department of Orthopaedics and Trauma Surgery University of Brescia School of Medicine, Viale Europa 11 25123, Brescia, Italy

**Keywords:** knee injury, posterior cruciate ligament bony avulsion, posterior cruciate ligament injury, suture anchor fixation

## Abstract

**Introduction:** The posterior cruciate ligament (PCL) is the largest and strongest intra-articular ligament of the knee joint and the primary posterior stabilizer. PCL injuries are less frequent than other knee ligament injuries and are typically combined with meniscal and chondral injuries or in the context of multiligamentous injuries. It is critical to properly diagnose and treat these lesions in order to avoid the risk of PCL insufficiency, subsequent knee instability, and early osteoarthritis. Surgical management can vary, and the ideal fixation device is still debated. Suture anchors are an unusual mean of fixation of PCL tibial bony avulsion. We report on two patients treated with open anchor fixation for PCL tibial bony avulsion with a follow-up of 3 years.

**Case Presentation:** A 15-year-old male and a 65-year-old male were treated with open anchor fixation for bony tibial avulsion of the PCL. Surgical treatment was performed at 5 weeks and 3 weeks after the trauma, respectively. Diagnosis was made with an X-ray followed by CT and MR scans. Repair was achieved by reinserting the PCL bony fragment to its posterior tibial eminence with suture anchors through an open posterior approach. Both patients recovered full knee stability and a pain-free full range of motion (ROM) within 4 months and returned to their previous activities with a high satisfaction. The patient has been followed up for 3 years, and no complications were observed.

**Conclusion:** PCL bony avulsions are rare, and their optimal treatment remains a significant subject of debate, particularly in the skeletally immature patient. We believe that open repair with metal anchors could be a good choice to repair PCL bony tibial avulsion in patients without concomitant intra-articular lesions and immature growth plates or severe fragmentation.

## 1. Introduction

The posterior cruciate ligament (PCL) is the largest and strongest intra-articular ligament of the knee joint and the primary posterior stabilizer. PCL injuries are less frequent than other knee ligament injuries [1] and are typically combined with meniscal and chondral injuries or in the context of multiligamentous injuries [2]. In most cases, these injuries are intrasubstance; however, although less common, femoral detachment and tibial avulsion have been described [3]. Tibial avulsions occur mainly in young, active patients, as a result of high-energy trauma, especially car and motorcycle accidents [4]. The mechanism of injury involves a force applied to the proximal tibia with the knee flexed. The incidence of tibial avulsions of the PCL is higher in countries with higher rates of motorcycle use [5] and more common than avulsion from its femoral bony attachment [6]. Knee hyperextension can also cause tibial avulsion of the PCL; this occurs most frequently in sports-related injuries [4]. In children, tibial avulsions are more common than intrasubstance PCL injuries because the ligament is stronger than its physeal attachment [7]. It is crucial to properly diagnose and treat these lesions in order to avoid the risk of PCL incompetence and subsequent knee instability and early osteoarthritis [8]. Diagnosis is achieved with plain X-rays of the knee in anteroposterior and lateral projection. PCL avulsion fractures appear as a focal discontinuity of the PCL facet at the posterior aspect of the tibia. If radiographs are insufficient, CT or MRI should be done. The Meyers and McKeever classification is used to grade these lesions: Type I injuries represent a minimally displaced avulsion, type II a hinged avulsion, and type III a completely detached avulsion [9]. Over the years, various techniques have been proposed to treat PCL tibial avulsions. Although it is clear that displaced fractures should be treated surgically, there is no consensus regarding the approach (arthroscopic or open) or the fixation device to be used [4, 10]. Suture anchors are an unusual mean of fixation of PCL tibial bony avulsion. We report on two patients treated with open anchor fixation for bony tibial avulsion of the PCL with 3-year follow-up.

## 2. Case Presentation: Patient 1

**Figure 1:**
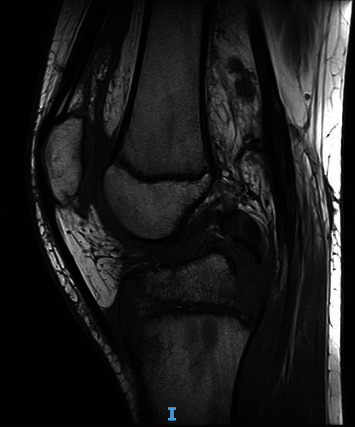
MRI of Case 1. The sagittal view shows PCL avulsion from the posterior tibial plateau. Growth cartilages are visible on both femur and tibia.

**Figure 2:**
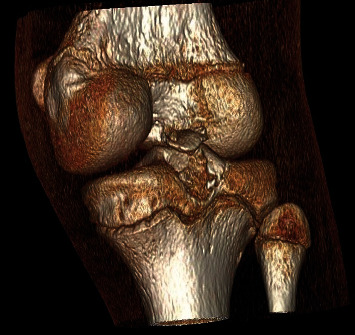
CT scan of Case 1. The 3D reconstruction shows the avulsed tibial fragment from the posterior tibial plateau.

**Figure 3:**
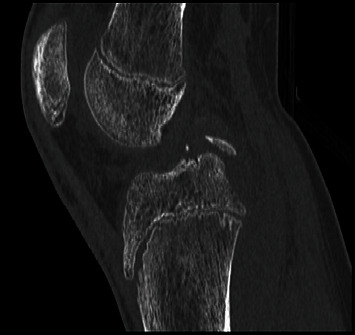
CT scan of case 1. The sagittal scan shows the displaced avulsed fragment.

**Figure 4:**
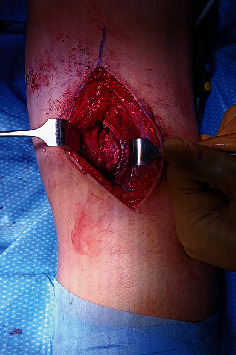
Open approach of case 1. The interval between the medial gastrocnemius and semimembranosus was developed. The neurovascular bundle was protected by the medial gastrocnemius, which was retracted laterally. The capsule was incised longitudinally and was exposed using two retractors to the posteromedial tibial plateau.

**Figure 5:**
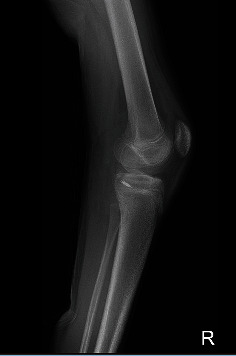
RX lateral view of Case 1. Metal anchor inserted at LCP tibial insertion.

**Figure 6:**
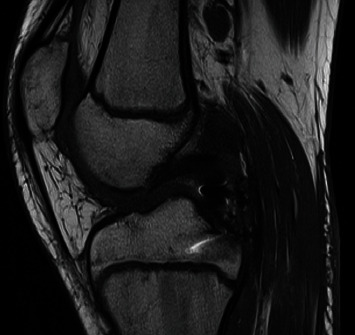
MRI of Case 1. The sagittal view 2 months after surgery shows the ligament inserted at its footprint but detached.

Informed consent was given by the patient for the publication of this report and associated figures. A 15-year-old male was admitted to the emergency department after a collision injury during motocross training. Emergency examination revealed a displaced diaphyseal fracture of the left ulna and a fracture of the V right metatarsal bone. The right knee presented with pain and significant joint effusion which proved to be blood following joint aspiration. Clinical examination showed a positive posterior drawer test with recurvation in extension with a range of motion (ROM) of +5° hyperextension (contralateral side: 0°) and full normal flexion. The Lachman test was negative, and there were no signs of injury to the structures of the posterolateral corner (as they are often concomitant with PCL tears) or to the menisci. Radiographs of the right knee showed an avulsion of the posterior tibial eminence. A CT and MRI confirmed the tibial bony avulsion of the LCP and ruled out concomitant meniscal or other ligamentous injury (Figures 1, 2, and 3). The ulna fracture was treated with open reduction and internal fixation while the metatarsal bone fracture was treated with cast immobilization. Open repair of LCP avulsion was scheduled at a later stage. Surgical treatment was performed 5 weeks after the injury, when the patient recovered a pain-free full knee ROM. The patient was placed in a prone position with the knee flexed to 30° in spinal anaesthesia. A tourniquet was inflated around the thigh. A gentle, inverted S incision was made along the flexion crease, beginning about 6 cm distal to the joint line, extending proximally, and then curving more horizontally to the popliteal crease. The interval between the medial gastrocnemius and semimembranosus was developed. The neurovascular bundle was protected by the medial gastrocnemius, which was retracted laterally. The capsule was incised longitudinally, and the posterior tibial eminence was exposed using retractors (Figure 4). The avulsed LCP bony fragment was identified and reinserted on its tibial footprint with a 5-mm metal anchor. One anchor was found to be sufficient to ensure correct tensioning and stability of ligament repair (Figure 5). A surgical drain was inserted, and the incision was closed in a layered fashion. The knee was placed into a hinged brace to prevent posterior tibial displacement. Postoperative protocol included thromboembolic prophylaxis and no weight bearing for 1 month. The knee brace was locked at 20° of flexion for 2 weeks to avoid active hamstring contraction which could move the tibia posteriorly, thus negatively affecting repair tension in the first phases of healing. The brace was unlocked by 30° each week to allow recovery of active and passive ROM. One month after surgery, gradual progressive weight bearing was allowed with the brace fully unlocked. Isometric quadriceps contraction and abduction/adduction exercises were initiated. Two months after surgery, the patient recovered full knee ROM and returned to walk. The patient did not report subjective instability although the posterior drawer test showed objective mild laxity. On MRI, the metallic anchor was visible well above the growth plate which had not been violated. The PCL appeared continuous and well tensioned (Figure 6). Three months after surgery, he returned to full training and competitive racing.

## 3. Case Presentation: Patient 2

**Figure 7:**
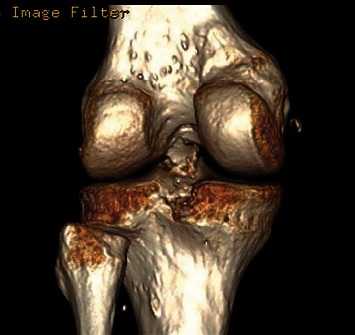
CT scan of Case 2. The 3D reconstruction shows the avulsed tibial fragment from the posterior tibial plateau.

**Figure 8:**
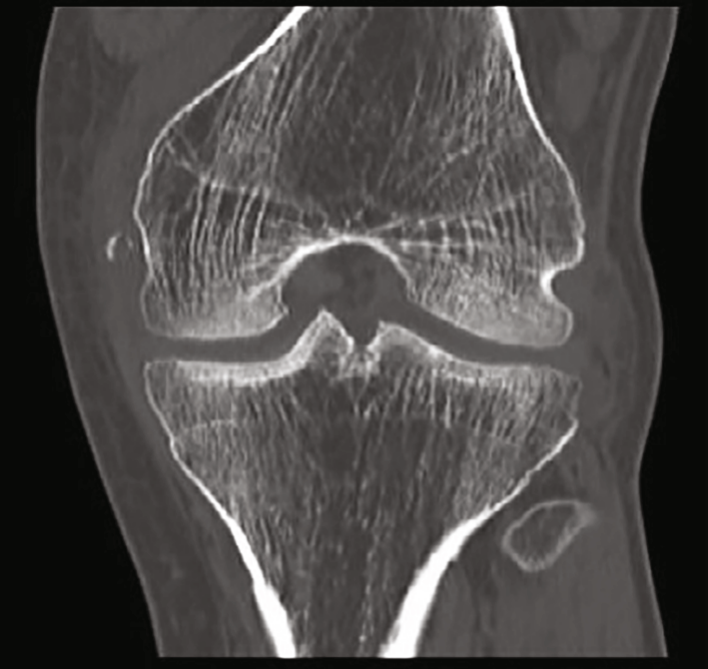
CT scan of Case 2. The coronal scan shows femoral Segond fracture at the proximal insertion of LCM.

**Figure 9:**
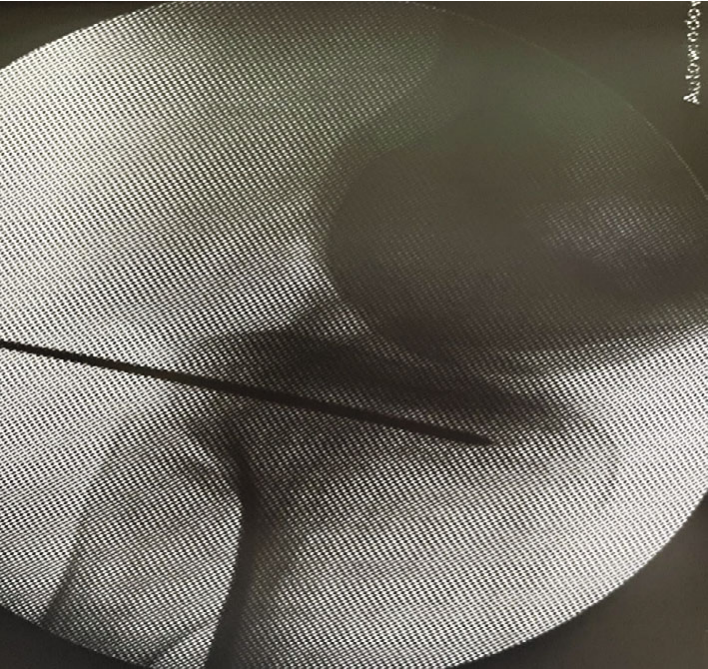
Intraoperative fluoroscopy of Case 2. Temporary stabilization with K-wire.

**Figure 10:**
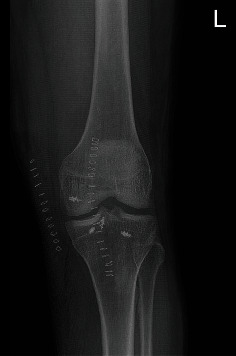
Postoperative A-P radiography of Case 2.

**Figure 11:**
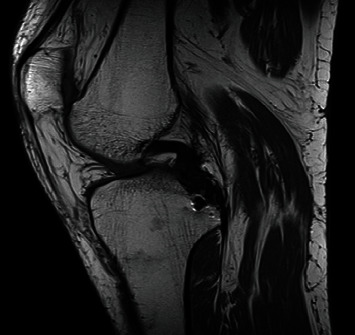
MRI at 2-year follow-up of Case 2. The LCP is inserted and continuous.

Informed consent was given by the patient for the publication of this report and associated figures. A 65-year-old man presented for orthopaedic assessment for joint effusion and left knee pain following a fall on a scooter a few days earlier which had not improved with painkillers. The knee showed signs of posterior pivot instability with a positive posterior drawer test but not posterolateral instability or injury to the posterolateral corner. Instead, valgus instability with pain on the femoral insertion of the medial collateral ligament (MCL) was found. Lachman's test and anterior drawer test were negative, as were meniscal tests. The patient underwent radiography of the knee with anteroposterior and latero-lateral views and an MRI that showed PCL bony tibial avulsion and posteromedial corner (PMC) injury (KD III M according to the Schenck classification of knee multiligament injuries) [11]. No meniscal lesions were found. CT scan with 3D reconstructions showed a comminuted LCP bony tibial avulsion and a bony femoral avulsion of the MCL (Figures 7 and 8). Surgical treatment was performed 3 weeks after the injury. The patient was placed in a prone position with the knee flexed to 30° in spinal anaesthesia. A tourniquet was inflated around the thigh. A posteromedial approach to the knee was performed to expose the posterior facet of the posterior tibial plateau. A posterior miniarthrotomy was performed to identify the avulsed articular fragments. The bony avulsion was severely comminuted so that it could not be fixed with screws. The main fragment was reduced and temporarily fixed using a 3-mm K-wire with fluoroscopic assistance (Figure 9). Subsequently, three 5-mm metal anchors were used to reinsert and properly tension the LCP back on its tibial footprint. A surgical drain was inserted, and the incision was closed in a layered fashion. The patient was then turned supine, and the MCL and the posteromedial capsule were repaired using a medial skin incision with a 5-mm double-stranded high-strength metal anchor (Figure 10). The knee was placed in a postoperative splint. The postoperative protocol included thromboembolic prophylaxis and no weight bearing for 35 days. The knee was placed in a hinged brace locked at 30° of flexion. The rehabilitation program included first passive then active joint recovery with hydrokinesis for maintenance of muscle trophism without loading. One month after surgery, the knee was stable with mildly limited flexion and full extension without recurvatum of the knee (ROM: 0°–100°). Four months after surgery, the patient recovered full ROM and resumed walking unassisted. Three years after surgery, the patient underwent a new MRI showing a continuous and well-tensioned PCL (Figure 11). The knee was stable, with no subjective or objective laxity. At this point, the patient had fully resumed his activities and was extremely satisfied.

## 4. Discussion

PCL avulsions are rare, and diagnosis can be difficult. However, if not treated properly, they can result in major morbidity. Often PCL avulsions occur in the context of high-energy trauma, so attention must be paid to any concomitant injuries. According to the Meyers and McKeever classification, type I injuries can be treated nonsurgically while type II and type III injuries should be treated surgically. In a recent paper, Yoon, Kim, and Park [12] proposed a nonsurgical treatment for avulsions with displacements of less than 6.7 mm. Other authors consider surgery the best option to ensure proper tensioning of the PCL, to shorten patient recovery time, and to avoid risks associated with prolonged immobilization [13]. Although there is currently no consensus, we believe that PCL avulsion fractures generally require surgical intervention. Operative treatment of PCL avulsion fractures achieves a high rate of fracture union with excellent restoration of posterior tibial translation [14]. Nonsurgical treatment is reserved for nondisplaced or minimally displaced avulsions in the absence of concomitant ligament injuries. Over the years, various treatments (open surgery and arthroscopic techniques) have been proposed, although there is no consensus regarding the optimal surgical management [15]. An open approach enables direct visualization of the fracture and better anatomical reduction, although it carries the risk of damaging the popliteal neurovascular bundle or the gastrocnemius muscle due to excessive retraction. Young patients are at risk of surgical wound abnormal healing [16, 17]. The main advantage of arthroscopic treatment is the possibility to simultaneously treat associated intra-articular lesions (such as meniscal tears) as well as facilitate patient recovery thanks to its less invasiveness. Hooper et al. [4] reported higher subjective and objective knee outcome scores following arthroscopic treatment. However, arthroscopic treatment does not allow direct visualization of the fragment thereby impairing anatomical reduction. This could result in malunion or residual posterior laxity. Compared to open surgery, arthroscopy is also technically more challenging and requires highly specialized equipment and expertise [18]. The most common complication reported in both open and arthroscopic techniques is arthrofibrosis, which occurs more frequently after arthroscopic procedures [4]. In both of our patients, there were no associated intra-articular lesions; therefore, we chose an open treatment instead of an arthroscopic one. Over the years, many fixation devices such as resorbable screws, staples, lag screws, suture anchors, cannulated screws, and toothed plate have been used [19, 20]. Recently, Guo et al. [13] proposed a new technique using a “homemade pin-hook.” The ideal fixing device must be minimally invasive, allow satisfactory reduction and stability, and be readily available. Until now, the debate on what devices may be of choice is still open. The size and degree of comminution of the avulsed fragment play a major role in the choice of the device: small bone fragments (< 10 mm) should be fixed with multiple suture anchors, although they might fail in case of severe comminution. Medium-sized bone fragments (10–20 mm) can be fixed by wires, and single large fragments (> 20 mm) can be fixed by cannulated screws [21]. In 2005, an arthroscopic technique with anchor fixation showed adequate tension and strength [22]. We believe that an open approach and the use of anchors provide the best method of repair. Our experience showed that treatment of tibial avulsions of the LCP with metal anchors can be safe and effective. Anatomic reduction by open surgery is recommended because it enables optimal tensioning of the ligament. Metal anchors avoid the need for fixation techniques, such as bone tunnels, that violate the growth plate in the skeletally immature patient. In our first case, there was mild objective residual posterior laxity although subjectively well tolerated. We believe that in patients with an immature skeleton, reinsertion with anchors could be a good choice because it provides knee stability without damaging the growth plate. Perhaps some patients could eventually undergo a second reconstructive surgery, but this can be safely delayed until complete skeletal growth takes place.

## 5. Conclusion

PCL bony avulsions are rare, and their optimal treatment continues to be controversial, especially in the skeletally immature patient. We believe that open repair with metal anchors could be a good option for treating PCL bony tibial avulsion in patients without concomitant intra-articular lesions and immature growth plates or severe fragment comminution.

## 6. Clinical Takeaway

Reinsertion should be preferable to reconstruction for PCL avulsion as it allows for a more anatomic repair.

Open surgery allows more accurate reinsertion of the avulsed PCL as it offers a better anatomical view and is technically much less demanding compared to arthroscopy achieving the same good outcomes.

Suture anchors are a good choice to repair PCL avulsion in the skeletally immature patient as they do not interfere with growth plates unlike screws or bone tunnel placement.

## Data Availability

Data supporting the findings of this study are available upon request to the corresponding author.
